# A construction and comprehensive analysis of ceRNA networks and infiltrating immune cells in papillary renal cell carcinoma

**DOI:** 10.1002/cam4.4309

**Published:** 2021-10-01

**Authors:** Yaqi Fan, Fangfang Dai, Mengqin Yuan, Feiyan Wang, Nanhui Wu, Mingyuan Xu, Yun Bai, Yeqiang Liu

**Affiliations:** ^1^ Shanghai Skin Disease Hospital Tongji University School of Medicine Shanghai China; ^2^ Department of Obstetrics and Gynecology Renmin Hospital of Wuhan University Wuhan Hubei China; ^3^ Shanghai Skin Disease Clinical College of Anhui Medical University Shanghai Skin Disease Hospital Shanghai China

**Keywords:** ceRNA network, immune cells, nomogram, PRCC

## Abstract

**Background:**

As the second most common malignancy in adults, papillary renal cell carcinoma (PRCC) has shown an increasing trend in both incidence and mortality. Effective treatment for advanced metastatic PRCC is still lacking. In this study, we aimed to establish competitive endogenous RNA (ceRNA) networks related to PRCC tumorigenesis, and analyze the specific role of differentially expressed ceRNA components and infiltrating immune cells in tumorigenesis.

**Methods:**

CeRNA networks were established to identify the key ceRNAs related to PRCC tumorigenesis based on the 318 samples from The Cancer Genome Atlas database (TCGA), including 285 PRCC and 33 normal control samples. The R package, “CIBERSORT,” was used to evaluate the infiltration of 22 types of immune cells. Then we identified the significant ceRNAs and immune cells, based on which two nomograms were obtained for predicting the prognosis in PRCC patients. Finally, we investigated the co‐expression of PRCC‐specific immune cells and core ceRNAs via Pearson correlation test.

**Results:**

COL1A1, H19, ITPKB, LDLR, TCF4, and WNK3 were identified as hub genes in ceRNA networks. Four prognostic‐related tumor‐infiltrating immune cells, including T cells CD4 memory resting, Macrophages M1, and Macrophages M2 were revealed. Pearson correlation test indicated that Macrophage M1 was negatively related with COL1A1 (*p* < 0.01) and LDLR (*p* < 0.01), while Macrophage M2 was positively related with COL1A1 (*p* < 0.01), TCF4 (*p* < 0.01), and H19 (*p* = 0.032). Two nomograms were conducted with favorable accuracies (area under curve of 1‐year survival: 0.935 and 0.877; 3‐year survival: 0.849 and 0.841; and 5‐year survival: 0.818 and 0.775, respectively).

**Conclusion:**

The study constructed two nomograms suited for PRCC prognosis predicting. Moreover, we concluded that H19‐miR‐29c‐3p‐COL1A1 axis might promote the polarization of M2 macrophages and inhibit M1 macrophage activation through Wnt signaling pathway, collaborating to promote PRCC tumorigenesis and lead to poor overall survival of PRCC patients.

## Graphical abstract

We constructed two nomograms for predicting the survival of papillary renal cell carcinoma patients.H19‐miR‐29c‐3p‐COL1A1 axis might promote the polarization of M2 macrophages and inhibit M1 macrophage activation through Wnt signaling pathway, collaborating to promote PRCC tumorigenesis and lead to poor overall survival of PRCC patients.

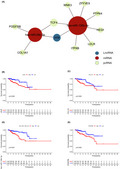

## 1 INTRODUCTION

As a type of malignant tumor, renal cell carcinoma (RCC) has exhibited increasing incidence and mortality in recent years, affecting over 400,000 individuals worldwide per year.^1^, ^2^ PRCC, a histological subtype of RCC, is characterized by the presence of papillae in local lesions.^3^ PRCC accounts for 15%–20% of RCC^4^, ^5^ and has become the second most common malignant renal tumor in adults.^6^ Although targeted therapies, including those targeting mTOR and VEGF, have been used for metastatic RCC clinically, PRCC patients displayed lower response rates compared with clear cell renal cell carcinoma (CCRCC) patients.^7^, ^8^ Previous studies have also reported that PRCC has different driver genes than CCRCC.^9^ As efficient therapy for terminal and metastatic PRCC is still lacking, new biomarkers are needed for diagnosis and treatment to ameliorate the overall survival (OS) of PRCC patients.

In competitive endogenous RNA (ceRNA) network, long non‐coding RNAs (lncRNAs) can competitively sequester microRNAs (miRNAs) from other targets, therefore post‐transcriptionally regulating the expression of genes and their biological functions.^10^ Increasing evidence indicates that imbalances in ceRNA networks play a pivotal part in the initiation and progression of malignancies.^11^, ^12^ In RCC, the low expression of lncRNA NBAT1 resulted in reducing the competitive inhibition of miR‐146 against GSK‐3β, facilitating cell proliferation and migration.^13^ A recent report revealed that upregulated miR‐1293 and miR‐3199‐2 might impact genes and predict a poor prognosis in PRCC.^14^ A ceRNA network also regulates the cross talk between tumor cells and immune cells.^10^ In tumors, infiltration of immune cells influences growth and metastasis. In PRCC, Linehan et al. reported that an increased proportion of type 2 T helper cells often indicated a poor prognosis.^3^ Compared with other pathological types of RCC, the response rate to the existing immune checkpoint inhibitors in PRCC is not satisfactory,^7^ so new PRCC‐specific diagnostic and prognostic immune checkpoints are required. Previous work found that upregulated LAG3 and PD‐L2 were significantly correlated with poor outcomes in PRCC.^15^ A recent study described a prognosis signature including 14 pairs of immune‐linked biomarkers for the prediction of PRCC prognosis.^16^ However, the co‐expression and regulatory function of ceRNAs and immune cells in PRCC has not yet been reported.

In this study, we analyzed RNA‐Seq profiling of PRCC from TCGA database, and established ceRNA networks related to tumorigenesis. CIBERSORT was applied to investigate the proportions of tumor‐related immune cells in the PRCC and control groups. Multivariate Cox and Lasso analyses were used; two nomograms were generated based on these analyses to predict the outcome in PRCC patients. Finally, we investigated and visualized the correlation between key ceRNAs and tumor‐related immune cells in an effort to identify novel molecules for prognosis prediction and clinical treatment of PRCC.

## 2 MATERIALS AND METHODS

### 2.1 Data collection and analysis of differentially expressed genes

This study obtained the consent of the Ethics Committee of Blinded per Author Guidelines. The RNA‐Seq v.2 and miRNA‐seq data of 285 PRCC and 33 normal control samples were downloaded from TCGA and standardized for subsequent analysis (Supplementary Tables S1 and S2). Clinical information was obtained from the Xena browser.^17^ Using R software and the “edgeR” package, we obtained differentially expressed RNAs, including lncRNAs, miRNAs, and mRNAs. When a *p* < 0.05, log2 (fold change) >1.0, or log2 (fold change) <−1.0 was considered statistically significant. We also performed Gene Ontology (GO) and Kyoto Encyclopedia of Genes and Genomes (KEGG) enrichment analyses of differentially expressed genes and visualized them using the R package “enrichplot” and “GOplot.”

### 2.2 Construction of ceRNA networks

We established ceRNA networks as follows: (a) differentially expressed lncRNAs, miRNAs, and genes were matched based on interactions in the StarBase database^18^; (b) successfully matched ceRNAs, exhibiting statistical significance (*p* < 0.01) in hypergeometric testing analysis and correlation analysis, were chosen as candidates for ceRNA networks. Cytoscape 3.8.2^19^ was applied to visualize ceRNA networks; and (c) experimentally validated miRNA–mRNA and lncRNA–miRNA interaction information was downloaded from miRTarBase (http://mirtarbase.mbc.nctu.edu.tw/)^20^ and Lncbase v.2 Experimental Module (http://carolina.imis.athena‐innovation.gr/diana_tools/web/index.php?r=lncbasev2%2Findex‐experimental).^21^ In the ceRNA candidate networks, the miRNA–mRNA and lncRNA–miRNA interaction pairs with experimental supporting data were screened as ceRNA network members, and Cytoscape 3.8.2 was used to draw the ceRNA network diagram.

### 2.3 Prognostic value of hub members in ceRNA networks and construction of nomogram

First, we employed Kaplan–Meier analysis for all members of the ceRNA networks to evaluate gene expression levels and prognostic performance for PRCC patients. Second, we integrated all components in the ceRNA networks into a Cox proportional hazards mode, and performed univariate Cox analysis to screen ceRNAs significantly related to OS and a Lasso regression model to avoid overfitting. Lastly, we formulated a nomogram based on multivariate Cox regression analysis to predict the outcome of PRCC patients in 1, 3, and 5 years. Last, we adopted receiver operating characteristic (ROC) and calibration curves to evaluate the accuracy and deviation of the nomogram.

### 2.4 Tumor‐infiltrating immune cells and co‐expression analysis

Based on the CIBERSORT algorithm (http://cibersort.stanford.edu/),^22^ we determined the infiltration proportions of 22 immune cells in PRCC and control groups. Samples with a *p *< 0.05 were outputted for subsequent analysis. Key immune cells that varied between the PRCC and control groups were picked via Wilcoxon rank‐sum test. Simultaneously, Kaplan–Meier analysis was used to estimate whether key immune cells could predict the prognosis. After Lasso regression adjustment, important immune cells were included in a Cox proportional hazard model. Next, we generated a nomogram for predicting outcomes of PRCC patients at 1, 3, and 5 years based on infiltration abundances of immune cells, followed by calibration and ROC curves to verify the performance of the nomogram. In the end, we used Pearson correlation analysis to investigate the co‐expression between hub ceRNAs and tumor‐infiltrated immune cells.

### 2.5 Multidimensional validation

To minimize bias, we employed multiple databases to verify our analysis results. CellMarker^23^ and String^24^ databases were applied to validate the interactive relationship between hub ceRNAs and specific markers on the surface of tumorigenesis‐related immune cells. Moreover, we searched the Oncomine database,^25^ OncomiR^26^ and GEPIA^27^ databases to explore expression levels of key ceRNAs and their clinical relevance in multiple tumor sets.

### 2.6 Statistical analysis and visualization

Only *p *< 0.05 on both sides were considered having a statistical significance. Samples with CIBERSORT output value *p* < 0.05 are considered eligible for further analysis. The Wilcoxon rank‐sum test was used to detect related immune cells. All analyses were operated by R 4.0.3 and R packages.

GDCRNATools,^28^ an R/Bioconductor package, was used for downloading RNA‐Seq data, ceRNAs regulatory network analysis, univariate survival analysis, and functional enrichment analysis.

edgeR was designed for the analysis of replicated count‐based expression data and is an implementation of methodology developed by Robinson and Smyth.^29^ To moderate the wise‐varied gene probes, edgeR models count data using an more complex overdispersed Poisson model, and uses an empirical Bayes procedure to moderate the degree of overdispersion across genes.^30^ In the present study, the pairwise exact testing procedure of differential analysis between tumor and normal groups was carried out using an exact test analogous to Fisher's exact test, but adapted for overdispersed data.

The survival difference was evaluated and visualized using Kaplan–Meier survival curves, and the association was tested via log‐rank tests.^31^

Ggplot2,^32^ a contributed visualization package, was used for visualizing the differentially expressed biomarkers. Corrplot^33^ was applied to draw bar plot and co‐expression figure. Pheatmap,^34^ vioplot, ggpubr,^35^ timeROC,^36^ RMS, and stringi were also employed to visualize the results.

## 3 RESULTS

### 3.1 Identification of differentially expressed genes

**FIGURE 1:**
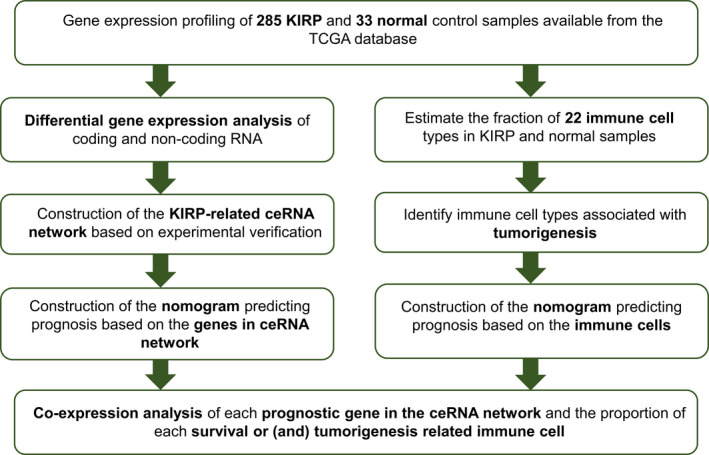
The analysis steps of this study

**FIGURE 2:**
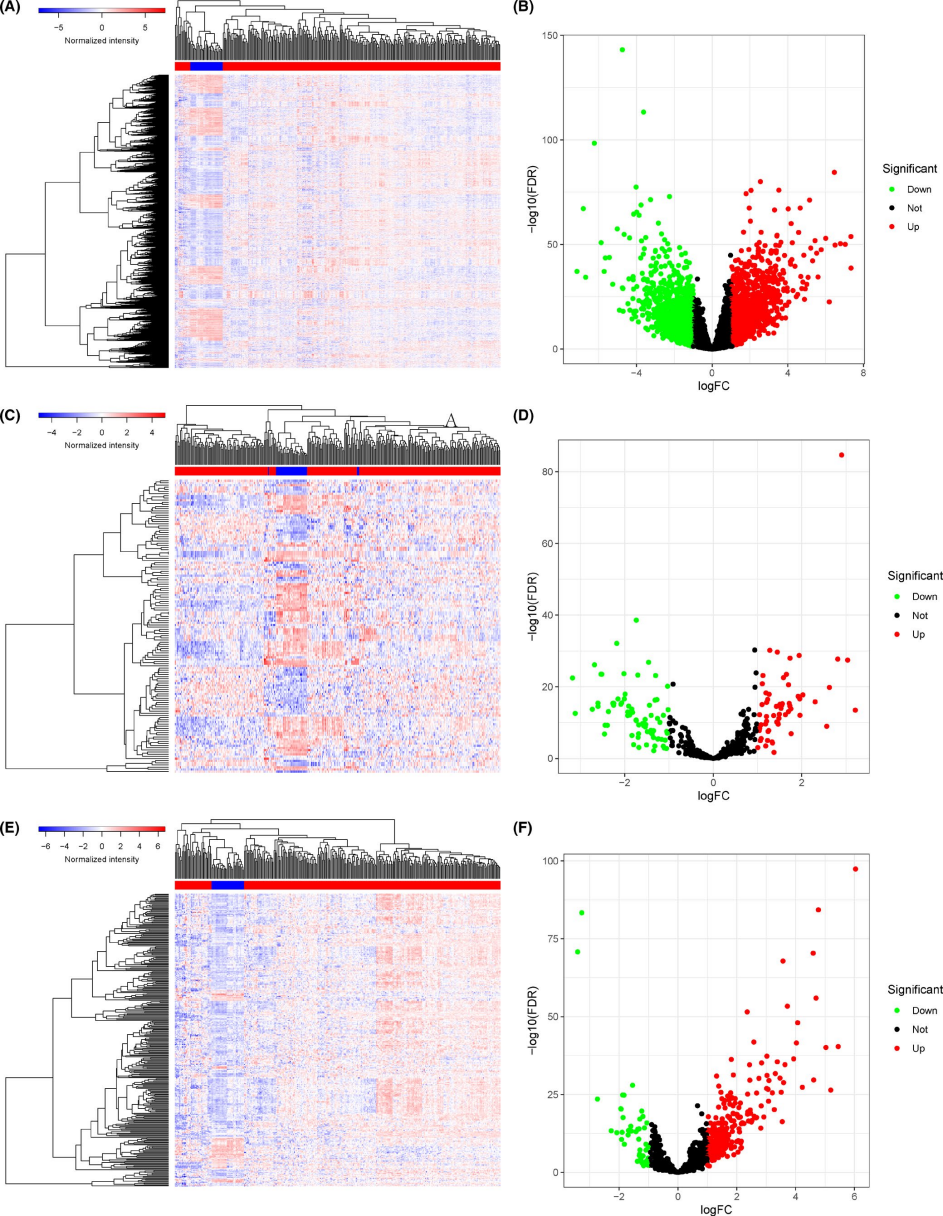
The differentially expressed genes in PRCC. The heatmap (A) and the volcano plot (B) of all differentially expressed mRNAs; the heatmap (C) and the volcano plot (D) of all differentially expressed miRNAs; the heatmap (E) and the volcano plot (F) of all differentially expressed lncRNAs. PRCC, papillary renal cell carcinoma; mRNAs, messenger RNAs; miRNAs, microRNAs; lncRNAs, long non‐coding RNAs

**FIGURE 3:**
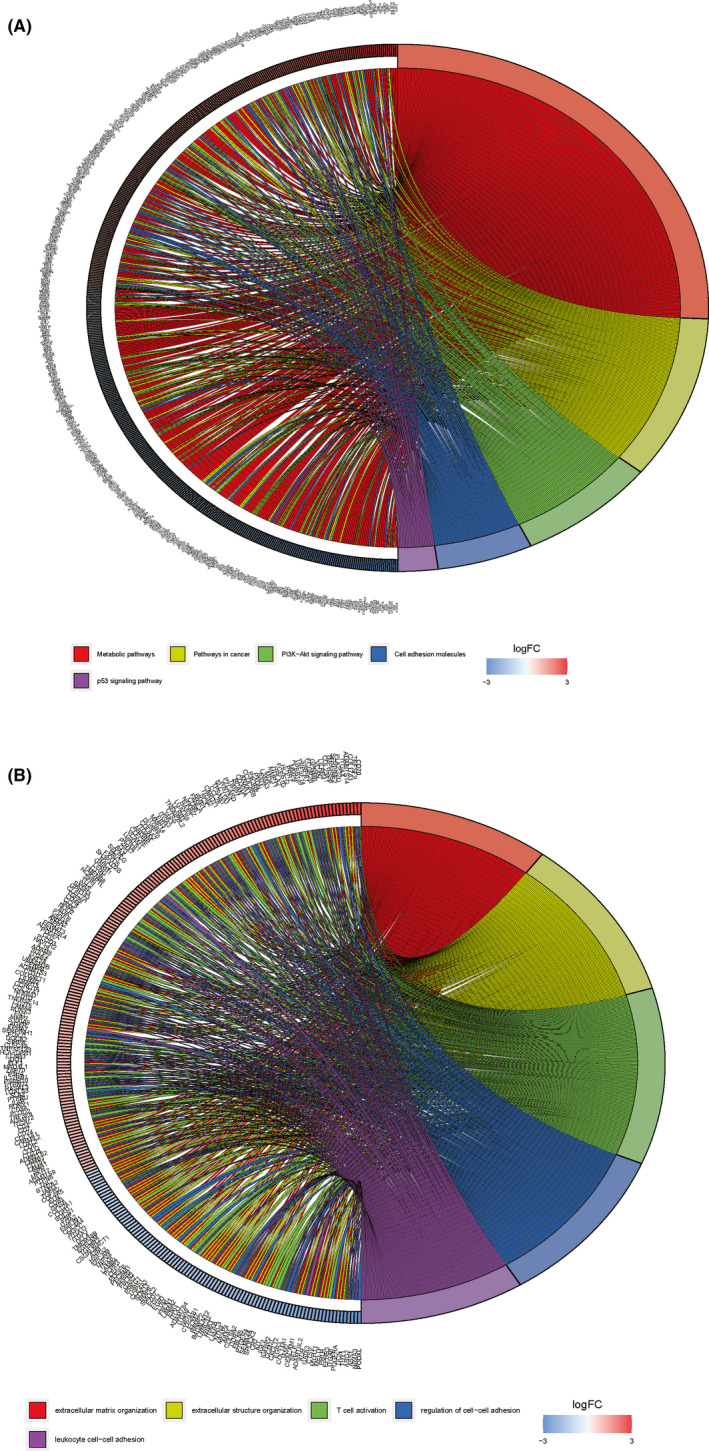
(A) Gene ontology function enrichment circle and (B) Pathway enrichment cycle of differentially expressed mRNAs

Figure 1 elucidates the analysis steps of our research. When defined log (fold change) >1.0 or <−1.0 and false discovery rate (FDR) <0.05 as the critical point, we obtained 2692 protein‐coding genes (1579 upregulated and 1113 downregulated), 136 miRNAs (59 upregulated and 77 downregulated), and 306 lncRNAs (259 upregulated and 47 downregulated) that expressed significantly different between PRCC and normal tissues (Figure 2A–F). GO and KEGG analyses of these differentially expressed RNAs (Figure 3A,B) indicated that the five most enriched GO terms were extracellular matrix organization (GO:0030198), extracellular structure organization (GO:0043062), T‐cell activation (GO:0042110), regulation of cell‐cell adhesion (GO:0022407), and leukocyte cell‐cell adhesion (GO:0007159). The top five pathways of gene enrichment were metabolic pathways (hsa01100), pathways in cancer (hsa05200), PI3K‐Akt signaling pathway (hsa04151), cell adhesion molecules (hsa04514), and p53 signaling pathway (hsa04115).

### 3.2 Establishment of the ceRNA networks and their prognostic value

**FIGURE 4:**
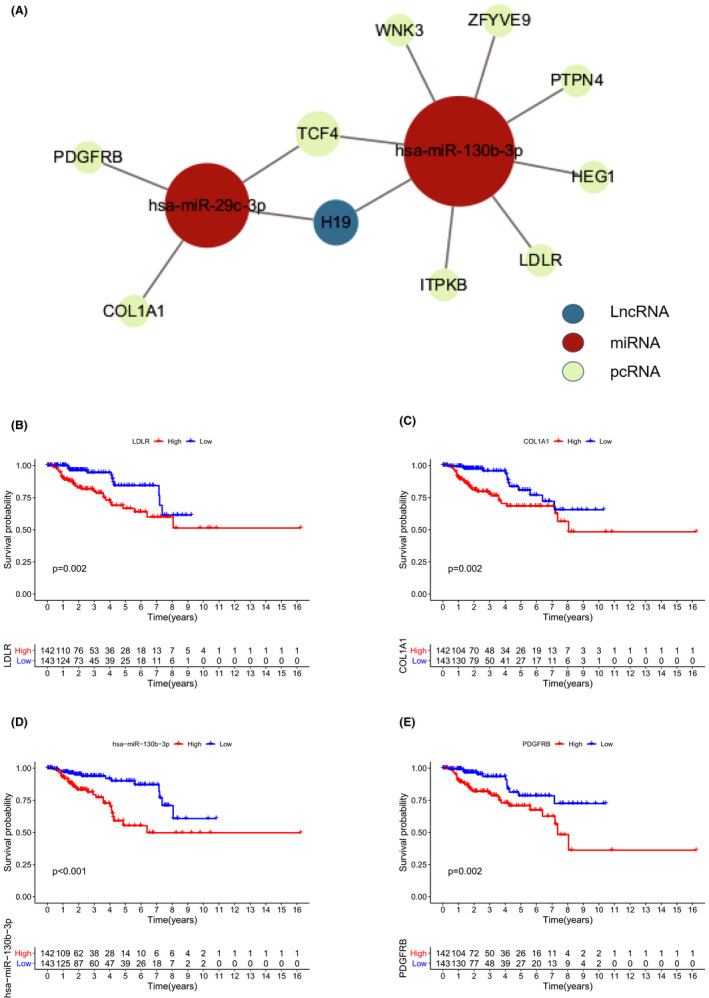
(A) The experimental validated ceRNAs networks. (B) LDLR, (C) COL1A1, (D) has‐miR‐130b‐3p, and (E) PDGFRB had significantly prognostic values

Through hypergeometric distribution and correlation tests (*p* < 0.01), we obtained the candidate network of ceRNAs, including 7 lncRNA–miRNA pairs and 50 miRNA–mRNA pairs (Supplementary Figure S1). We further removed RNA pairs lacking experimental supports, resulting in a final ceRNA network containing 1 lncRNA, 2 miRNAs, and 10 protein‐coding genes. The only lncRNA, H19, regulated the function of miR‐29c‐3p and miR‐130b‐3p by competitive binding, affecting the inhibition of 10 downstream genes and regulating related pathways (Figure 4A, Table 1). In Kaplan–Meier analysis, the biomarkers LDLR (*p *= 0.002), COL1A1 (*p *= 0.002), hsa‐miR‐130b‐3p (*p *< 0.001), and PDGFRB (*p *= 0.002), exhibited pertinence for the prognosis (Figure 4B–E).

**TABLE 1:** Results of hypergeometric testing and correlation analysis of H19‐related ceRNAs networks

PcRNA	MiRNAs	Correlation *p*	Hypergeometric test *p*
LDLR	hsa‐miR‐130b‐3p	<0.001	0.0025
PTPN4	hsa‐miR‐130b‐3p	<0.001	0.0036
ZFYVE9	hsa‐miR‐130b‐3p	<0.001	0.0005
ITPR1	hsa‐miR‐130b‐3p	0.0003	0.0012
WNK3	hsa‐miR‐130b‐3p	0.0017	0.0002
TCF4	hsa‐miR‐29c‐3p, hsa‐miR‐130b‐3p	<0.001	0.0003
PDGFRB	hsa‐miR‐29c‐3p	<0.001	0.0045
HEG1	hsa‐miR‐29c‐3p,	<0.001	0.0042
COL1A1	hsa‐miR‐29c‐3p	0.0002	0.0042

CeRNA, competing endogenous RNA; pcRNA, protein‐coding RNA; miRNA, microRNA.

### 3.3 Construction of the prediction model based on hub genes in ceRNA networks

**FIGURE 5:**
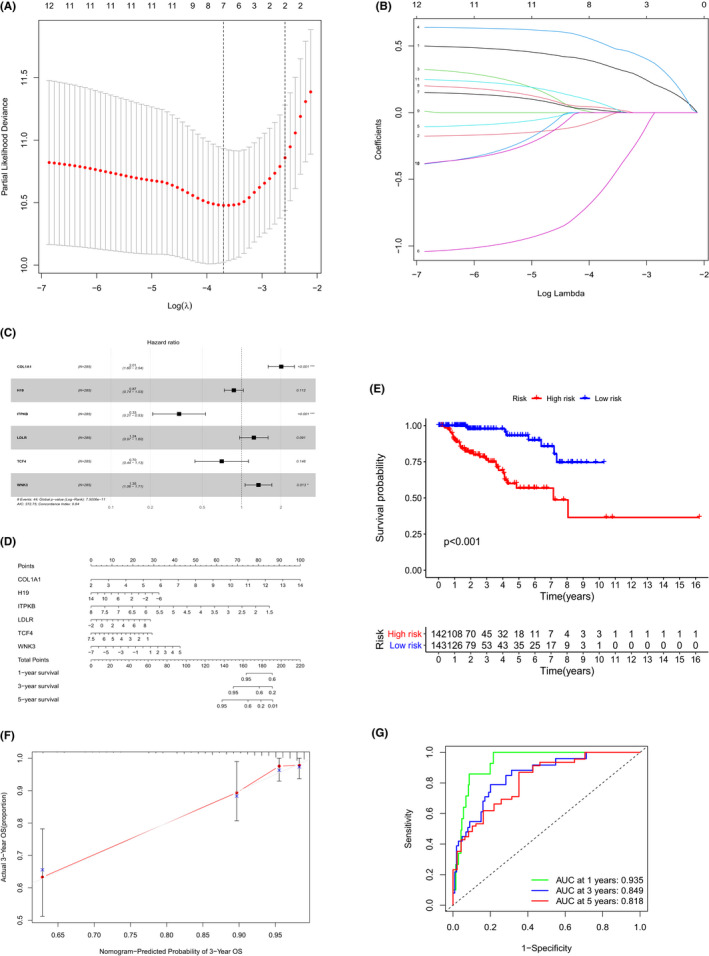
(A, B) Through lasso regression analysis, six molecules (COL1A1, H19, ITPKB, LDLR, TCF4, and WNK3) were integrated into the (C) Cox proportional hazards model. (D) Nomogram based on the Cox hazard model for predicting the OS of PRCC patients. (E) RNAs in the panel (C) exhibited survival capability. (F) Calibration curve and (G) ROC curve for suggesting the prediction ability and accuracy of nomogram. The area under curve of 1‐year, 3‐year, and 5‐year survival was 0.935, 0.849, and 0.818, respectively. PRCC, papillary renal cell carcinoma; ROC, receiver operating characteristic

Ten genes and three non‐coding RNAs in the ceRNA networks were included in univariate Cox regression and Lasso regression analyses (Figure 5A,B), and six molecules (COL1A1, H19, ITPKB, LDLR, TCF4, and WNK3) were found to be critical for prognostic modeling (Akaike information criterion [AIC] = 372.75). Among them, COL1A1, WNK3, and ITPKB were statistically remarkable risk factors of PRCC (Table 2). Subsequently, the six genes were included in a Cox proportional hazards model to assess their prognostic abilities (Figure 5C). Based on the Cox proportional hazards model, we generated a nomogram to suggest OS probability of PRCC patients in the future 1, 3, and 5 years (Figure 5D; concordance index, 0.840). Based on the risk score of predictive multivariate Cox regression model,^37^ division of the PRCC samples into a high‐risk group and low‐risk group resulted in significantly different OS (*p* < 0.001, Figure 5E). The ROC curve suggested accuracy of nomogram (AUC of 1‐year survival: 0.935; 3‐year survival: 0.849; and 5‐year survival: 0.818). Besides, calibration curve suggested an acceptable discrimination of the nomogram (Figure 5F,G).

**TABLE 2:** The significant components of the ceRNA networks included in the Cox proportional hazards model for overall survival in PRCC patients

Gene	Hazard ratio	95% CI		*p* value
		Lower	Upper	
COL1A1	2.01	1.60	2.54	<0.001***
ITPKB	0.33	0.21	0.53	<0.001***
H19	0.87	0.74	1.32	0.112
LDLR	1.24	0.97	1.60	0.091
WNK3	0.70	1.06	1.71	0.013*
TCF4	1.34	0.44	1.13	0.146

CeRNAs, competing endogenous RNAs; PRCC, papillary renal cell carcinoma; CI, confidence interval.

*p < 0.05; ***: *p* < 0.001.

### 3.4 Proportions of PRCC‐related immune cells

**FIGURE 6:**
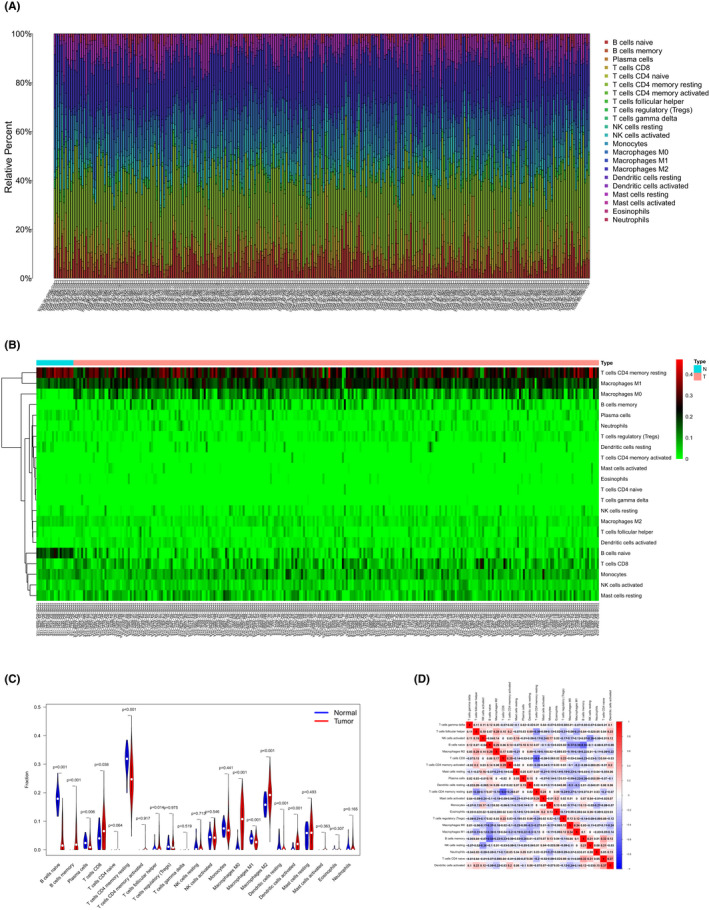
(A) Bar plot showing immune cell types and relative percent in PRCC tissues. (B) Heatmap of tumor‐infiltrating cells in PRCC tissues and control group tissues. On the top, the blue annotation represents the normal control group, and the red annotation represents PRCC patients. (C) Violin plot by Wilcoxon rank‐sum test for comparison of cells’ proportion between the normal and PRCC tissues. It revealed that the proportions of the B cells naive (*p* < 0.001), plasma cells (*p *= 0.006), resting memory CD4T cells (*p* < 0.001), M1 macrophages (*p* < 0.001), and resting dendritic cells (*p* < 0.001) in PRCC were reduced, and memory B cells (*p* < 0.001), CD8T cells (*p *= 0.038), follicular helper T cells (*p *= 0.014), M0 Macrophages (*p* < 0.001), M2 Macrophages (*p* < 0.001), and activated dendritic cells (*p* < 0.001) were increased in PRCC tissues. (D) Correlation analysis of different tumor‐infiltrating cells in PRCC samples. PRCC, papillary renal cell carcinoma

Using the CIBERSORT algorithm, we found the infiltration of M0 macrophages and naïve B cells was significantly different in PRCC and control samples (Figure 6A,B). Further evaluation of the different immune cell proportions in PRCC and control samples by Wilcoxon rank‐sum test (Figure 6C) revealed that the proportions of the B cells naive (*p* < 0.001), plasma cells (*p *= 0.006), resting memory CD4T cells (*p *< 0.001), M1 macrophages (*p* < 0.001), and resting dendritic cells (*p* < 0.001) in PRCC were reduced, and memory B cells (*p *< 0.001), CD8T cells (*p *= 0.038), follicular helper T cells (*p *= 0.014), M0 Macrophages (*p* < 0.001), M2 Macrophages (*p* < 0.001), and activated dendritic cells (*p *< 0.001) were increased in PRCC tissues. Finally, co‐expression analysis illustrated the complex relationships of 22 immune cell types (Figure 6D).

### 3.5 Clinical significance of PRCC‐infiltrating immune cells

**FIGURE 7:**
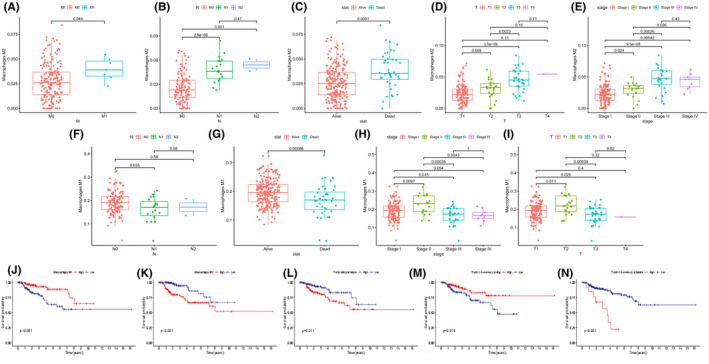
Relevance of 22 immune cells’ types with clinic and prognosis of PRCC. (A‐E) The proportion of M2 macrophage differed between M0 and M1 (*p* = 0.044), N0 and N1 (*p* < 0.0001), alive and dead (*p* = 0.0001), T1 and T3 (*p* < 0.0001), and stage I and stage III (*p* < 0.0001). (F‐I) The proportion of M1 macrophages differed between N1 and N0 (*p* = 0.035), T3 and T2 (*p* = 0.0004), dead and alive (*p* = 0.0009), and stage III and stage I (*p* = 0.0004). (J‐N) Kaplan–Meier analysis indicated that resting memory CD4T cells (*p* = 0.019) and M1 macrophages (*p* < 0.001) were associated with favorable survival status while activated memory CD4T cells (*p* < 0.001), Tfh cells (*p* = 0.011), and M2 macrophages (*p* < 0.001) were linked with poor survival status. PRCC, papillary renal cell carcinoma

We analyzed the importance of 22 types of infiltrated immune cells for clinical and prognosis prediction of PRCC. There were significantly more M2 macrophages presented between M0 and M1 (*p *= 0.044), N0 and N1 (*p* < 0.0001), alive and dead (*p *= 0.0001), T1 and T3 (*p* < 0.0001), and stage I and stage III (*p* < 0.0001) (Figure 7A–E). The proportion of M1 macrophages differed between N1 and N0 (*p *= 0.035), T3 and T2 (*p *= 0.0004), dead and alive (*p *= 0.0009), and stage III and stage I (*p *= 0.0004) (Figure 7F–I). Follicular helper T cells (Tfh) infiltrated differently between alive and dead (*p *= 0.0031), M0 and M1 (*p *= 0.026), stage I and stage III (*p *= 0.0017), and N0 and N1 (*p* < 0.0001) (Supplementary Figure S2A–D). Kaplan–Meier analysis of 22 types of immune cells illustrated that resting memory CD4T cells (*p *= 0.019) and M1 macrophages (*p* < 0.001) were associated with favorable survival status while activated memory CD4T cells (*p* < 0.001), Tfh cells (*p *= 0.011), and M2 macrophages (*p* < 0.001) were linked with poor survival status (Figure 7J–N).

### 3.6 Construction of the prediction model based on PRCC‐infiltrating immune cells

**FIGURE 8:**
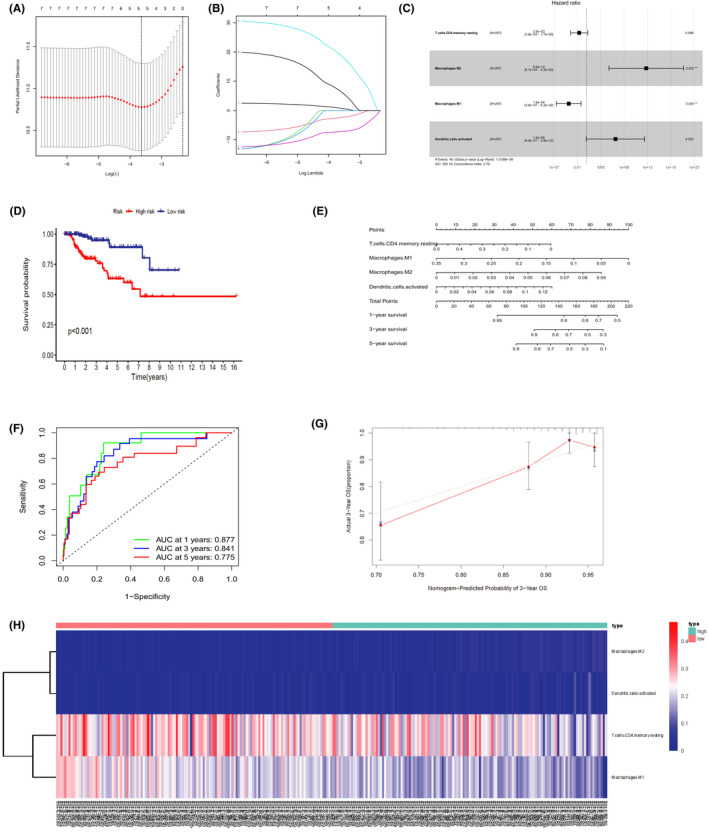
Based on (A, B) lasso regression, four immune cells, resting memory CD4T cells, M1 macrophages, M2 macrophages, and activated dendritic cells were included in (C) the multivariate Cox risk regression model. (D) Kaplan–Meier analysis based on the Cox regression model exhibited prognostic capability (*p* < 0.001). (E) Nomogram based on the Cox regression model for outcome prediction of PRCC patients. (F) ROC and (G) calibration curves suggested excellent accuracy and discrimination of the nomogram. The area under curve of 1‐year, 3‐year, and 5‐year survival is 0.877, 0.841, and 0.775. (H) A heatmap visualized the infiltration of four tumor‐related immune cells in the low‐risk score and high‐risk score groups. PRCC, papillary renal cell carcinoma; ROC, receiver operating characteristic

The results of lasso regression (Figure 8A,B) indicated that four immune cells, resting memory CD4T cells, M1 macrophages, M2 macrophages, and activated dendritic cells were suitable for modeling and were integrated into a multivariate Cox risk regression model (Table 3). Hazard ratio is calculated (Figure 8C). The multivariate Cox regression model displayed a significant difference in survival status when the tumor samples were divided into high‐ and low‐risk groups (Figure 8D, *p* < 0.001). Based on the multivariate Cox model, we fabricated a nomogram for predicting the prognosis of PRCC patients at future 1, 3, and 5 years (concordance index, 0.790; Figure 8E). The ROC and calibration curves illuminated the excellent predictive accuracy (AUC of 1‐, 3‐, and 5‐year survival: 0.877, 0.841, and 0.775, respectively) and discrimination of the nomogram (Figure 8F,G). Figure 8H shows the infiltration levels of resting memory CD4T cells, M1 macrophages, M2 macrophages, and activated dendritic cells in the low‐risk score and high‐risk score groups.

**TABLE 3:** The significant infiltrated immune cells included in the Cox proportional hazards model for overall survival in PRCC patients

Immune cells	Hazard ratio	95% CI		*p* value
		Lower	Upper	
T cells CD4 memory resting	2.5e−02	3.8e−04	1.7e+00	0.086
Macrophages M2	5.6e+12	6.1e+04	5.2e+20	0.002**
Macrophages M1	1.5e−04	3.6e−07	6.3e−02	0.004**
Dendritic cells activated	1.5e+06	9.0e−01	2.6e+12	0.052

KRCC, papillary renal cell carcinoma; CI, confidence interval.

**p < 0.01.

### 3.7 Co‐expression relationship between immune cells and key genes

**FIGURE 9:**
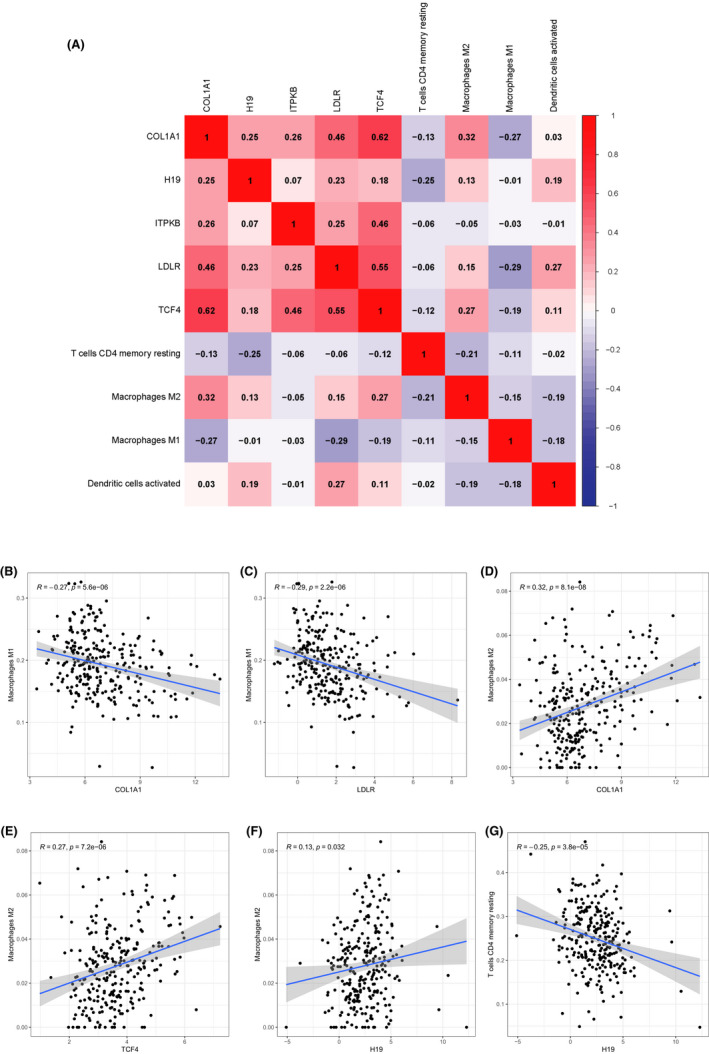
Co‐expression analysis of immune cells and key genes by Pearson's correlation coefficient. (A) The co‐expression heatmap of immune cells and key genes. (B) M1 macrophages were negatively associated with (B) COL1A1 (*R* = −0.27, *p* < 0.001) and (C) LDLR (*R* = −0.29, *p* < 0.001). M2 macrophages were positively associated with (D) COL1A1 (*R* = 0.32, *p* < 0.001), (E) TCF4 (*R* = 0.27, *p* < 0.001), and (F) H19 (*R* = 0.13, *p* = 0.032). (G) Resting memory CD4T cells and H19 were negatively correlated (*R* = −0.25, *p* < 0.001)

We adopted Pearson's correlation analysis to explore the co‐expression relationship between key genes in the ceRNA network and the prognosis‐related immune cells (Figure 9A). As shown in Figure 9, M1 macrophages were negatively associated with COL1A1 (*R* = −0.27, *p* < 0.001, Figure 9B) and LDLR (*R* = −0.29, *p* < 0.001, Figure 9C) while M2 macrophages were positively associated with COL1A1 (*R* = 0.32, *p* < 0.001, Figure 9D), TCF4 (*R* = 0.27, *p* < 0.001, Figure 9E), and H19 (*R* = 0.13, *p *= 0.032, Figure 9F). Resting memory CD4T cells and H19 were negatively correlated (*R* = −0.25, *p* < 0.001, Figure 9G).

### 3.8 Multidimensional validation

To further reveal the association between key ceRNAs and PRCC‐related immune cells, we constructed an interaction network of proteins including cell markers of M2 macrophages (CD163, CD206, CCL18, CD209, FIZZ1, and IL‐10 from the CellMarker database), cell markers of M1 macrophages (CD16, CD32, CD64, CD68, CD80, CD86, and CXCL10 from the CellMarker database), and prognosis‐related genes (COL1A1, ITPKB, LDLR, and PDGFRB) (Supplementary Figure S3). GEPIA was used to explore the relationship between COL1A1, M1 macrophage, and M2 macrophage surface markers, and the co‐expression of COL1A1 with CD64, CD80, CD206, and CD209 was observed (Supplementary Figure S4A–D, *p* < 0.001).

In the Oncomine and GEPIA databases, COL1A1 expression was upregulated in a variety of tumors, and its expression in multiple PRCC sequencing datasets (GSE15641 and GSE2109) was markedly higher compared with normal kidney tissues (*p* < 0.05). It was also correlated with the pathological stages of PRCC patients (Supplementary Figure S5A–D). LDLR expression was decreased in two datasets (GSE15641 and GSE2109) and was significantly correlated with the pathological stages of PRCC patients (Supplementary Figure S6A–D). Additionally, according to OncomiR, miR‐29c‐3p was correlated with tumorigenicity of 16 kinds of tumors, along with a pronounced association with clinical M stage and pathological M and T stages of PRCC patients (Supplementary Tables S3 and S4).

## 4 DISCUSSION

As a kidney malignancy, the incidence of PRCC is constantly increasing.^38^ mTOR‐ and VEGF‐targeted therapies have become the most promising treatments for advanced metastatic RCC; however, PRCC patients exhibit poor response rates. There still remains a demand for identification of novel biomarkers and immune targets for improved treatment of patients with advanced PRCC. The cross talk between ceRNA networks and the infiltration of immune cells plays a crucial part in regulating tumor progression. The present research reported differences in RNA expression and infiltrating immune cells between tumor and normal samples, and revealed pathways for enrichment of differentially expressed genes. In RCC, the p53‐mediated mitochondrial pathway was found to induce tumor apoptosis.^39^ Based on a previous report, activation of the PI3K‐AKT pathway was associated with the epithelial–mesenchymal transition and proliferation, migration, and invasion of tumor cells.^40^ We also screened ceRNAs and immune‐infiltrating cells related to tumorigenicity and prognosis, and generated two nomograms for outcome prediction.

Through bioinformatics analysis, we set up tumorigenesis‐related ceRNA networks composed of nine pairs of mRNAs–miRNAs–lncRNAs verified by experiments. Based on hypergeometric tests and correlation analysis, hsa‐miR‐29c‐3p (miRNA), COL1A1 (protein‐coding RNA), and H19 (lncRNA) were significantly correlated (*p *= 0.0002). Moreover, COL1A1 was negatively associated with M1 macrophage infiltration and positively related with infiltrating M2 macrophage while H19 was positively linked with M2 macrophage infiltration. According to the above results, we speculated that the tumorigenesis‐related ceRNAs H19, hsa‐miR‐29c‐3p, and COL1A1 and tumorigenesis‐related M1 and M2 macrophages might play crucial parts in PRCC tumorigenesis and progression.

As one of the earliest reported lncRNAs, H19 is located in human chromosome 11p15.5, and its RNA mainly exists in the cytoplasm and functions as a regulatory RNA or ribose regulator.^41^ In pancreatic cancer, H19 was reported to bind and inhibit miR‐194 to relieve the inhibition of PFTK1, thus activating Wnt signal transduction and increasing the proliferation and migration of tumor cells.^42^ H19 has also been shown to act as an miRNA sponge in colorectal, gastric, and bladder cancers, further regulating tumor progression.^43^, ^44^, ^45^ In RCC, Wang et al. demonstrated that lncRNA H19 was overexpressed in tumor tissues and correlated with tumor stage, lymph node metastasis, and distant metastasis.^46^ In CCRCC, the overexpression of lncRNA H19 was proved to promote the carcinogenesis of tumor.^47^ Besides, He et al. demonstrated that H19 involved in the migration and invasion of CCRCC by competitively sponging endogenous miR‐29a‐3p and regulating E2F1 expression.^48^ However, there are no further studies on correlation between PRCC and H19. In our analysis, we first reported that the lncRNA H19 was overexpressed in PRCC, and formed ceRNA networks together with downstream miRNAs (miR‐29c‐3p and miR‐130b‐3p) and their target genes (COL1A1, PTPN4, ITPK1, LDLR, TCF4, WNK3, ZFYVE9, PDGFRB, and HEG1). Hence, we speculated that H19 might act as an oncogene in PRCC, contributing to cancer development. MiR‐29c‐3p has been confirmed to suppress the progression of melanoma, ovarian cancer, breast carcinoma, and colorectal cancer, consistent with our results.^49^, ^50^, ^51^, ^52^ Through bioinformatics analysis, we found that H19 could regulate the expression of miR‐29c‐3p. We observed that miR‐29c‐3p was downregulated in PRCC and its downstream gene, COL1A1, was upregulated in PRCC tissues, which was consistent with previous studies.^53^ These results together suggested that H19 promoted PRCC tumorigenesis by competitively binding miR‐29c‐3p to alleviate inhibition of COL1A1, which has recently been shown to act as an oncoprotein in oral squamous cell carcinoma, gastric cancer, and ovarian cancer.^54^, ^55^, ^56^, ^57^, ^58^ We also found that COL1A1 was significantly related with OS and clinical TNM stage, which was determined to be an independent risk factor for PRCC oncogenesis (hazard ratio =2.01, *p* < 0.001). Joanna et al. compared RCC with normal tissues by RT‐PCR and found that COL1A1 expression was upregulated; this was linked with poor survival.^56^ Dysregulation of COL1A1 in RCC may be related to epigenetic activation, as COL1A1 has been reported to display promoter methylation in RCC tumor cell line DNA, but not in normal cell DNA.^59^ Previous researches have reported that COL1A1 could promote colorectal carcinoma by regulating the Wnt/PCP signaling pathway. Thus, COL1A1 might play a tumor‐promoting role in PRCC by activating Wnt signaling.^60^

In tumor microenvironment, macrophages are usually featured as M1‐ and M2‐polarized subtypes according to the differentiation. In this study, we observed that infiltration of M1 macrophages in tumor tissues was significantly lower than that in the normal tissues (*p* < 0.001), while that of M2 macrophages showed the opposite (*p* < 0.001). M1 and M2 macrophages were both associated with clinical stage, pathological T stage, and N stage. High levels of M1 macrophage infiltration are usually linked with good prognosis, and high‐proportioned M2 macrophages could signify poor outcomes. M1 macrophages can kill tumor cells and produce pro‐inflammatory cytokines; they can also promote apoptosis, inhibit tumorigenicity,^61^ and tumor phagocytosis.^62^ However, M2 macrophages usually regulate the inflammatory response and adaptive Th1 immunity, and facilitate angiogenesis, extracellular matrix remodeling, and repair, thus promoting tumorigenesis.^63^, ^64^ Our results further suggested that the infiltration of M1 and M2 macrophages could be a valuable prognostic indicator of PRCC outcome. The transformation of M2 macrophages to M1 might be a potential target for tumor treatment.

A study of gastric carcinoma showed that activation of the H19‐miR‐29a‐3p‐COL1A2 axis was positively correlated with M2 macrophage infiltration.^65^ A recent study found that H19 could be induced by M2‐type tumor‐associated macrophages, which was followed by activation of downstream ceRNA targets.^66^ These results are consistent with the co‐expression of M2 macrophages and H19, as well as M2 macrophages and COL1A1 in our study. We speculated that the H19‐miR‐29c‐3p‐COL1A1 axis was closely connected with the polarization of macrophages in PRCC. H19 and COL1A1 activated Wnt signaling, and were positively related with pathway‐associated proteins. A previous report indicated that Wnt signaling was involved in the regulation of macrophage polarization.^67^ Hub molecules in the Wnt signaling pathway were highly expressed in M2 macrophages, and Wnt pathway activator inhibited the activation of M1‐type macrophages.^68^ In PRCC patients, we found that the infiltration of M2 macrophages increased while the infiltration of M1 macrophages decreased. The H19‐miR‐29c‐3p‐COL1A1 axis might promote the polarization of M2 macrophages and inhibit the activation of M1 macrophages via Wnt signaling. Ultimately, this process could promote tumorigenicity and lead to a poor outcome of PRCC.

There are some limitations of our study. First, the databases used (TCGA‐KIRP, GSE15641, and GSE2109) are all from the west and lack multi‐species diversity. Second, the location of immune cell infiltration in the tumor is important for metastasis and prediction of therapeutic effect. Our analysis of immune cell infiltration lacked spatial properties. Still, this study yielded certain important results. We generated two nomograms for the prediction of OS in PRCC patients, and revealed that the H19‐miR‐29c‐3p‐COL1A1 axis was significantly associated with M1 and M2 macrophages polarization as well as tumorigenesis and prognosis of patients with PRCC; this could be useful for prognostic prediction and/or as a therapeutic target. Future work will further explore the effects of the H19‐miR‐29C‐3P‐COL1A1 axis on PRCC phenotypes, investigate the synergy of the H19‐miR‐29C‐3P‐COL1A1 axis and M1‐ and M2‐type macrophages in oncogenesis, and locate cells with high expression of COL1A1 and H19 in the single‐cell RNA‐Seq data for PRCC tissue.

## 5 CONCLUSION

In this study, two nomograms suitable for prognostic prediction of PRCC were constructed by analyzing hub members in ceRNA networks and the infiltrating immune cells related to PRCC tumorigenesis, which displayed favorable accuracy. The comprehensive predictive models are expected to improve individualized management of patients with advanced PRCC. Furthermore, the cross talk between the H19‐miR‐29c‐3p‐COL1A1 axis and the infiltration of M1 and M2 macrophages regulated PRCC development.

## CONFLICT OF INTEREST

There is no conflict of interest.

## AUTHOR CONTRIBUTIONS

Yeqiang Liu, Yaqi Fan, Fangfang Dai, and Mengqin Yuan designed the experiment and performed the most part of analysis. Yun Bai provided writing assistance. Feiyan Wang, Nanhui Wu, and Mingyuan Xu worked at information collection.

## ETHICAL APPROVAL

All procedures performed in studies involving human participants were in accordance with the ethical standards of the institutional and/or national research committee and with the 1964 Helsinki declaration and its later amendments or comparable ethical standards.

## Supporting information

Fig S1‐4

Fig S5

Fig S6

Table S1‐4

## Data Availability

Publicly available datasets were used in this research. We downloaded the dataset TCGA‐KIRP from GDC data portal (https://portal.gdc.cancer.gov).
