# Supplementary figures and images for: A construction and comprehensive analysis of ceRNA networks and infiltrating immune cells in papillary renal cell carcinoma
Source: Cancer Med. 2021 Oct 1;10(22):8192–209. doi: 10.1002/cam4.4309 (PMC8607257; doi:10.1002/cam4.4309)

S1

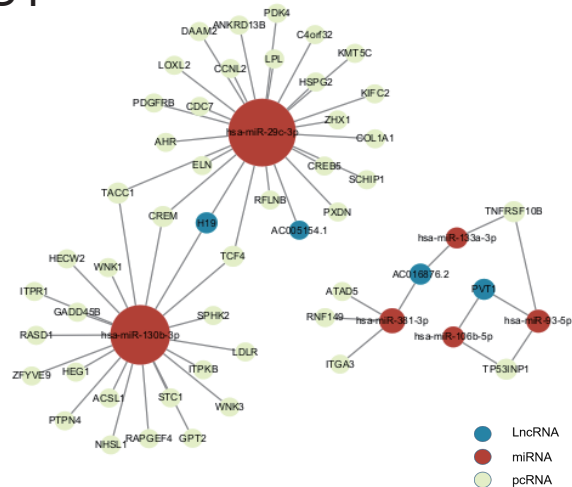

S2

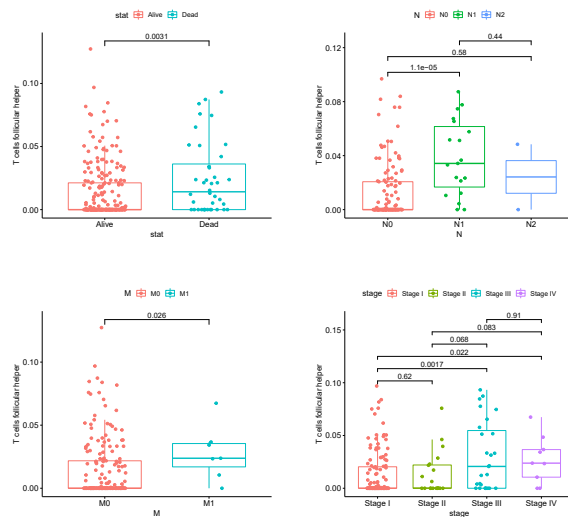

S3

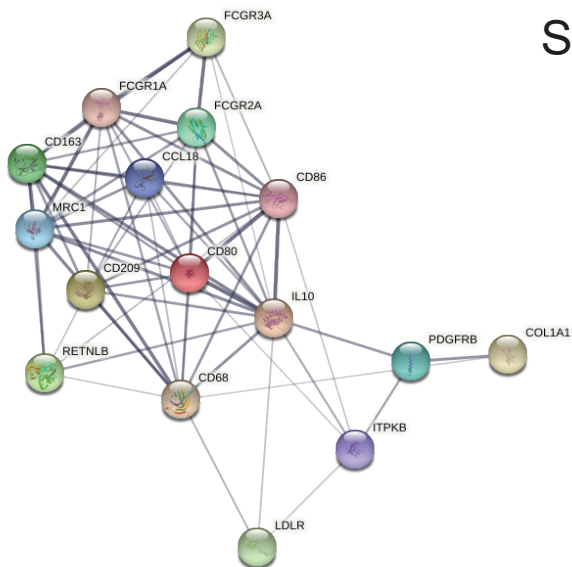

S4

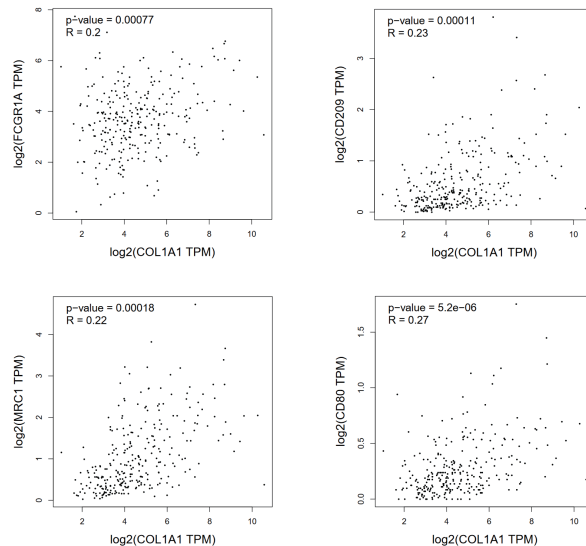

Supplement: Supplementary file 1 — Fig S1‐4 [file CAM4-10-8192-s003.pdf]
