# Supplementary material for: A construction and comprehensive analysis of ceRNA networks and infiltrating immune cells in papillary renal cell carcinoma
Source: Cancer Med. 2021 Oct 1;10(22):8192–209. doi: 10.1002/cam4.4309 (PMC8607257; doi:10.1002/cam4.4309)
Supplement: Supplementary file 2 — Fig S5 [file CAM4-10-8192-s002.pdf]

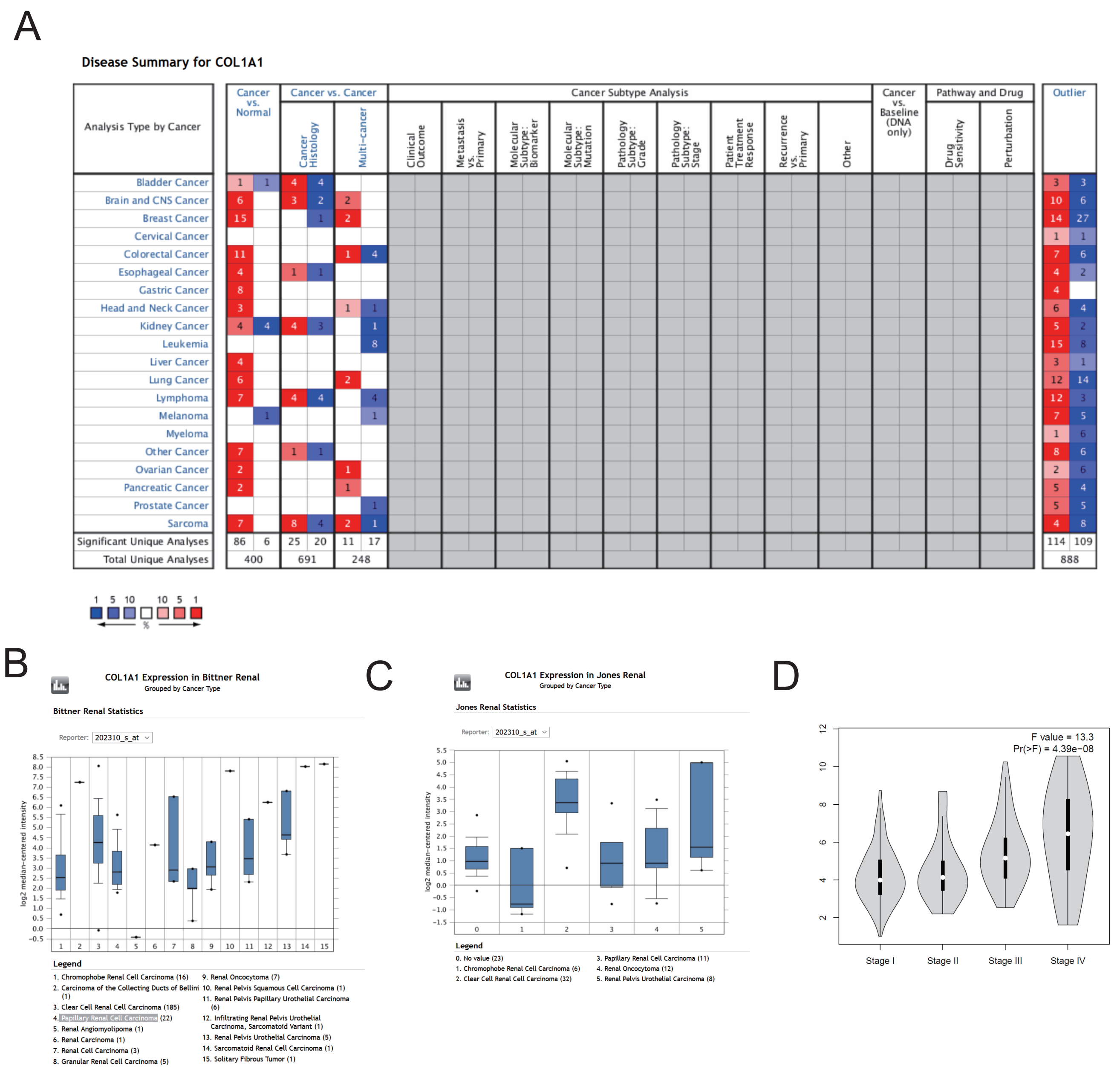
A
Disease Summary for COL1A1
Analysis Type by Cancer
Cancer vs. Normal
Cancer vs. Cancer
Cancer Histology
Multi-cancer
Cancer Subtype Analysis
Clinical Outcome
Metastasis vs. Primary
Molecular Subtype: Biomarker
Molecular Subtype: Mutation
Pathology Subtype: Grade
Pathology Subtype: Stage
Patient Treatment Response
Recurrence vs. Primary
Other
Cancer vs. Baseline (DNA only)
Pathway and Drug
Drug Sensitivity
Perturbation
Outlier
Bladder Cancer 1 1 4 4 3 3
Brain and CNS Cancer 6 3 2 2 10 6
Breast Cancer 15 1 2 14 27
Cervical Cancer 1 1
Colorectal Cancer 11 1 4 7 6
Esophageal Cancer 4 1 1 4 2
Gastric Cancer 8 4
Head and Neck Cancer 3 1 1 6 4
Kidney Cancer 4 4 4 3 1 5 2
Leukemia 8 15 8
Liver Cancer 4 3 1
Lung Cancer 6 2 12 14
Lymphoma 7 4 4 4 12 3
Melanoma 1 1 7 5
Myeloma 1 6
Other Cancer 7 1 1 8 6
Ovarian Cancer 2 1 2 6
Pancreatic Cancer 2 1 5 4
Prostate Cancer 1 5 5
Sarcoma 7 8 4 2 1 4 8
Significant Unique Analyses 86 6 25 20 11 17 114 109
Total Unique Analyses 400 691 248 888
1 5 10 10 5 1
%
B
COL1A1 Expression in Bittner Renal
Grouped by Cancer Type
Bittner Renal Statistics
Reporter: 202310_s_at
log2 median-centered intensity
Legend
1. Chromophobe Renal Cell Carcinoma (16)
2. Carcinoma of the Collecting Ducts of Bellini (1)
3. Clear Cell Renal Cell Carcinoma (185)
4. Papillary Renal Cell Carcinoma (22)
5. Renal Angiomyolipoma (1)
6. Renal Carcinoma (1)
7. Renal Cell Carcinoma (3)
8. Granular Renal Cell Carcinoma (5)
9. Renal Oncocytoma (7)
10. Renal Pelvis Squamous Cell Carcinoma (1)
11. Renal Pelvis Papillary Urothelial Carcinoma (6)
12. Infiltrating Renal Pelvis Urothelial Carcinoma, Sarcomatoid Variant (1)
13. Renal Pelvis Urothelial Carcinoma (5)
14. Sarcomatoid Renal Cell Carcinoma (1)
15. Solitary Fibrous Tumor (1)
C
COL1A1 Expression in Jones Renal
Grouped by Cancer Type
Jones Renal Statistics
Reporter: 202310_s_at
log2 median-centered intensity
Legend
0. No value (23)
1. Chromophobe Renal Cell Carcinoma (6)
2. Clear Cell Renal Cell Carcinoma (32)
3. Papillary Renal Cell Carcinoma (11)
4. Renal Oncocytoma (12)
5. Renal Pelvis Urothelial Carcinoma (8)
D
F value = 13.3
Pr(>F) = 4.39e−08
Stage I
Stage II
Stage III
Stage IV
