# Supplementary material for: A construction and comprehensive analysis of ceRNA networks and infiltrating immune cells in papillary renal cell carcinoma
Source: Cancer Med. 2021 Oct 1;10(22):8192–209. doi: 10.1002/cam4.4309 (PMC8607257; doi:10.1002/cam4.4309)

A

Disease Summary for LDLR

Analysis Type by Cancer	Cancer vs. Normal		Cancer vs. Cancer: Cancer Histology		Cancer vs. Cancer: Multi-cancer		Cancer Subtype Analysis: Clinical Outcome	Metastasis vs. Primary	Molecular Subtype: Biomarker	Molecular Subtype: Mutation	Pathology Subtype: Grade	Pathology Subtype: Stage	Patient Treatment Response	Recurrence vs. Primary	Other	Cancer vs. Baseline (DNA only)	Pathway and Drug: Drug Sensitivity	Perturbation	Outlier	
Bladder Cancer		1	1	1															3	2
Brain and CNS Cancer	1	1	1																15	14
Breast Cancer	1	6				1													19	28
Cervical Cancer	1																		2	3
Colorectal Cancer	2				1														13	13
Esophageal Cancer					1															4
Gastric Cancer																			4	3
Head and Neck Cancer						1													11	7
Kidney Cancer		4	3	4		2													9	4
Leukemia	2	1	6	4															29	16
Liver Cancer		1																	3	1
Lung Cancer		9		1															13	17
Lymphoma	4		1																16	12
Melanoma	2																		8	12
Myeloma																			5	4
Other Cancer				1															17	11
Ovarian Cancer			1																10	4
Pancreatic Cancer					4														3	2
Prostate Cancer																			8	10
Sarcoma			5	1		1													11	7
Significant Unique Analyses	13	23	16	12	6	4													182	161
Total Unique Analyses	442		746		260														976	

1 5 10 10 5 1
%

B

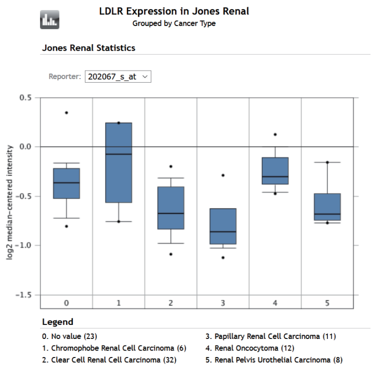

C

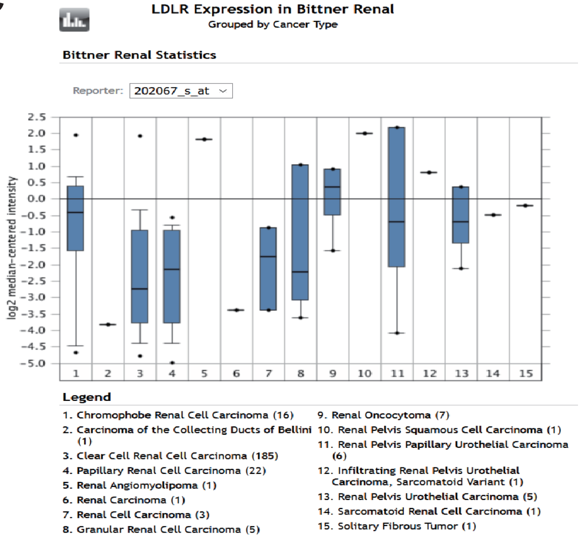

D

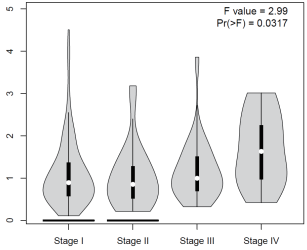

Supplement: Supplementary file 3 — Fig S6 [file CAM4-10-8192-s004.pdf]
