# Supplementary material for: A construction and comprehensive analysis of ceRNA networks and infiltrating immune cells in papillary renal cell carcinoma
Source: Cancer Med. 2021 Oct 1;10(22):8192–209. doi: 10.1002/cam4.4309 (PMC8607257; doi:10.1002/cam4.4309)
Supplement: Supplementary file 4 — Table S1‐4 [file CAM4-10-8192-s001.docx]

**Supplementary Table 1.** Baseline information of 285 patients diagnosed with kidney renal papillary cell carcinoma.

| **Variables** | **Total Patients (N = 285)** |
| --- | --- |
| **Age, years** |  |
| Mean ± SD | 61.32 ± 13.93 |
| **Gender** |  |
| Female | 76 (26.67%) |
| Male | 209 (73.33%) |
| **Race** |  |
| White | 203 (71.23%) |
| Asian | 6 (2.10%) |
| Black or African American | 60 (21.05%) |
| American indian or alaska native | 2 (1.08%) |
| Unknown | 14 (4.91%) |
| **Tissue type** |  |
| Tumor tissue only | 252 (88.42%) |
| Tumor tissue and nomal tissue | 33 (11.58%) |
| **Histological subtype** |  |
| Kidney renal papillary cell carcinoma | 285 (100%) |
| **Recurrence** |  |
| Yes | 21 (7.37%) |
| No | 138 (48.42%) |
| Unknown | 126 (44.21%) |

Abbreviations: SD: Standard deviation.

**Supplementary Table 2.** Baseline information of 33 patients providing para-cancer tissue.

| **Variables** | **Total Patients (N = 285)** |
| --- | --- |
| **Age, years** |  |
| Mean ± SD | 64.21 ± 12.02 |
| **Gender** |  |
| Female | 15 (45.45%) |
| Male | 18 (54.55%) |
| **Race** |  |
| White | 25 (75.76%) |
| Asian | 0 (0%) |
| Black or African American | 5 (15.15%) |
| American indian or alaska native | 1 (3.03%) |
| Unknown | 2 (6.06%) |
| **Survival status** |  |
| Alive | 24 (72.73%) |
| Dead | 9 (27.27%) |

Abbreviations: SD: Standard deviation.

**Supplementary Table 3.** OncomiR database indicated that the expression of miR-29c-3p was significantly associated with tumorigenesis of 16 types of cancers.

| **miRNA Name** | **Cancer Abbreviation** | **Upregulated in:** | **T- test P value** |
| --- | --- | --- | --- |
| hsa-miR-29c-3p | BRCA | Normal | 6.75e-03 |
| hsa-miR-29c-3p | CHOL | Normal | 3.29e-03 |
| hsa-miR-29c-3p | COAD | Tumor | 2.40e-02 |
| hsa-miR-29c-3p | ESCA | Normal | 3.38e-03 |
| hsa-miR-29c-3p | HNSC | Normal | 2.57e-12 |
| hsa-miR-29c-3p | KICH | Normal | 2.30e-04 |
| hsa-miR-29c-3p | KIRC | Normal | 9.75e-04 |
| hsa-miR-29c-3p | KIRP | Normal | 5.21e-07 |
| hsa-miR-29c-3p | LIHC | Normal | 4.55e-08 |
| hsa-miR-29c-3p | LUAD | Tumor | 1.98e-02 |
| hsa-miR-29c-3p | LUSC | Normal | 5.96e-13 |
| hsa-miR-29c-3p | PRAD | Normal | 5.31e-03 |
| hsa-miR-29c-3p | READ | Tumor | 3.96e-02 |
| hsa-miR-29c-3p | STAD | Normal | 3.68e-03 |
| hsa-miR-29c-3p | THCA | Normal | 1.45e-12 |
| hsa-miR-29c-3p | UCEC | Normal | 9.73e-06 |

Abbreviations: BRCA: breast invasive carcinoma; CHOL: cholangiocarcinoma; COAD: colon adenocarcinoma; ESCA: esophageal carcinoma; HNSC: head and neck squamous cell carcinoma; KICH: kidney chromophobe; KIRC: kidney renal clear cell carcinoma; KIRP: kidney renal papillary cell carcinoma; LIHC: liver hepatocellular carcinoma; LUAD: lung adenocarcinoma; LUSC: lung squamous cell carcinoma; PRAD: prostate adenocarcinoma; READ: rectal adenocarcinoma; STAD: stomach adenocarcinoma; THCA: thyroid carcinoma; UCEC: uterine corpus endometrial carcinoma

**Supplementary Table 4.** OncomiR database indicated that the expression of miR-29c-3p was significantly associated with clinical parameters of KIRP.

| **miRNA Name** | **Cancer Abbreviation** | **Clinical Parameters** | **ANOVA P-value** |
| --- | --- | --- | --- |
| hsa-miR-29c-3p | KIRP | Clinical M Status | 8.36e-01 |
| hsa-miR-29c-3p | KIRP | Pathologic M Status | 6.15e-01 |
| hsa-miR-29c-3p | KIRP | Pathologic T Status | 7.48e-01 |

Abbreviations: KIRP: kidney renal papillary cell carcinoma
